# Chemical Antagonists of Plasminogen Activator Inhibitor-1: Mechanisms of Action and Therapeutic Potential in Vascular Disease

**DOI:** 10.4172/1747-0862.1000125

**Published:** 2014-09-21

**Authors:** Tessa M Simone, Stephen P Higgins, Craig E Higgins, Michelle R Lennartz, Paul J Higgins

**Affiliations:** Center for Cell Biology & Cancer Research, Albany Medical College, Albany, New York 12208, USA

## Introduction

**Figure 1:**
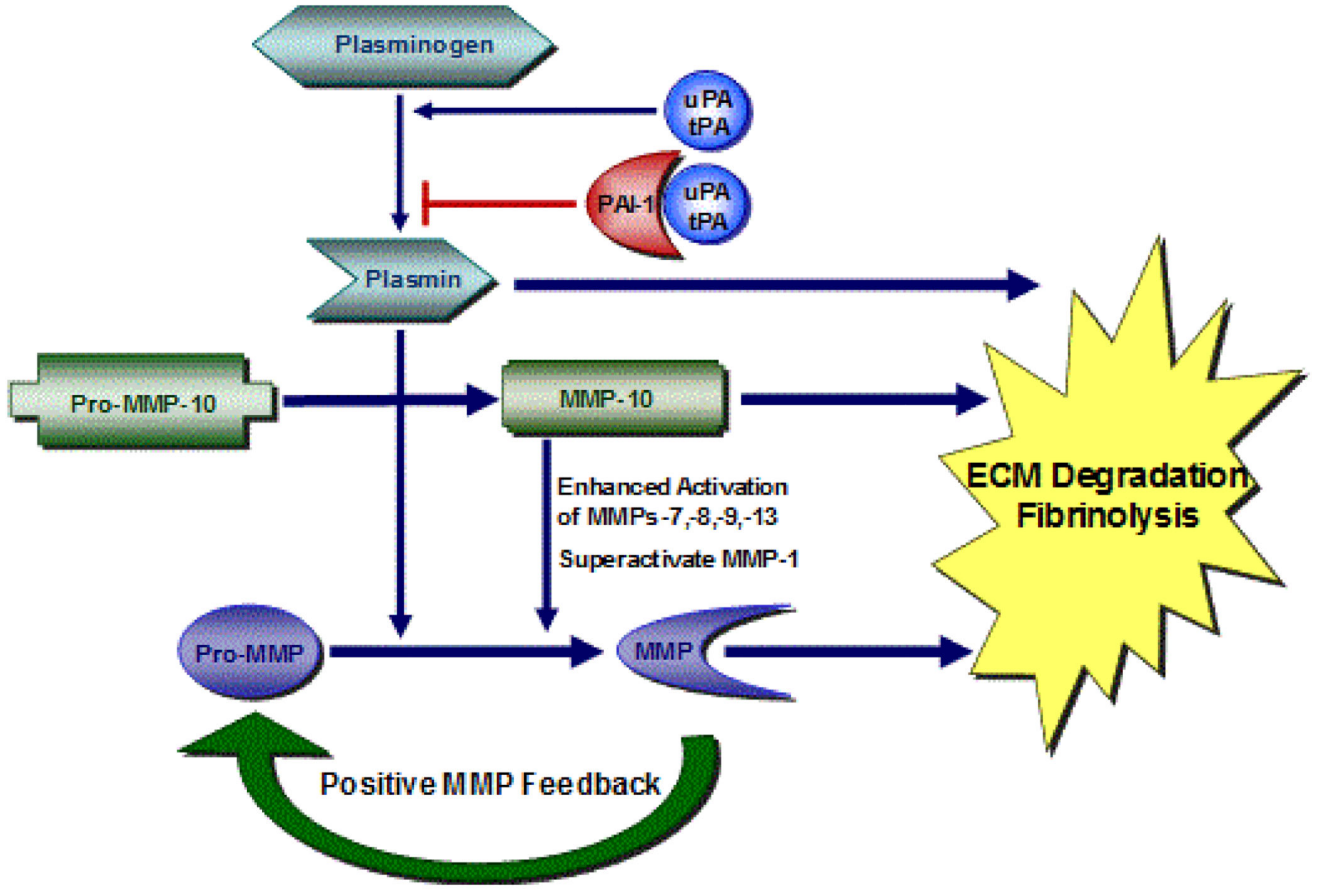
Regulation of the proteolytic microenvironment. A highly-interactive plasmin-MMP, pericellular proteolytic cascade is finely “titrated” both temporally and spatially by PAI-1. This cooperating system of proteases and inhibitors is fundamental to tissue repair and development of chronic diseases.

Plasminogen activator inhibitor-1 (PAI-1; SERPINE1), a clade E1 member of the serine protease inhibitor (SERPIN) superfamily, is a major inhibitor of urokinase (uPA) and tissue-type (tPA) plasminogen activators. By limiting the conversion of plasminogen to plasmin, PAI-1 attenuates fibrinolysis, promotes extracellular matrix (ECM) accumulation and contributes to both physiologic and pathophysiologic stromal remodeling (Figure 1). Cooperation between the plasmin-generating and matrix metalloproteinase (MMP) systems is normally closely controlled by a balance between individual proteases and their respective inhibitors. Dysregulation of one or more members in this cascade frequently accompanies chronic disorders and anomalies of the repair response. Elevated PAI-1 levels are, in fact, a significant causative factor in the pathophysiology of diabetes, vascular thrombosis, metabolic syndrome, septic coagulopathy, atherosclerosis, restenosis and myocardial infarction, particularly in the context of increased tissue TGF-β1 levels [1-4].

This review focuses on the role of PAI-1 in vascular disease and summarizes current evidence that pharmacologic blockade of PAI-1 function with small molecule inhibitors may have clinical utility as an anti-fibrotic modality. Indeed, oral administration of the PAI-1 inhibitor TM5275 effectively attenuates adenoviral-delivered TGF-β1 - induced pulmonary fibrosis, stimulated myofibroblast apoptosis and suppressed TGF-β1 -mediated expression of specific pro-fibrotic genes (e.g., fibronectin, PAI-1) [5].

## PAI-1 Structure/Function

PAI-1 is a single-chain, glycosylated protein, comprised of three β-sheets (A, B, C) and nine α-helical domains (A-I) with a strained reactive center loop (RCL) positioned in the carboxy terminus. Inhibition of protease activity occurs by formation of a covalent ester bond between the carboxyl group of Arg346 in the RCL of PAI-1 and the hydroxyl group of the active site serine in the protease target, mimicking the normal substrate-to-proteinase interaction, followed by formation of a reversible Michaelis-like 1:1 stoichiometric complex with its paired proteinase [6,7]. PAI-1 is termed a “suicide inhibitor” as it is rendered inactive by cleavage at the peptide bond (P1-P1’) in the RLC upon covalent complexing with the engaged protease [8,9].

PAI-1 is unique relative to other SERPINs as it exists in the structurally and functionally distinct active, latent and cleaved conformations [10,11]. PAI-1 is initially synthesized in an active but unstable state (half-life approximately 2 hours at 37°C, pH 7.4) and converts spontaneously into the latent form. Latency requires insertion of the N-terminus of the PAI-1 RCL into β-sheet A forming a new β-strand (s4A) which creates an unusual loop structure and conformational change in the reactive site, disrupting the peptide bond between Arg346 and Met347 (P1-P1’) ultimately preventing PAI-1 from interacting with proteinases [12-14]. Alternatively, PAI-1 can be cleaved by target proteases at the peptide bond (P1-P1’) without formation of a covalent complex thereby acting as a “substrate”. This cleavage causes the N-terminus of the RCL to insert into β-sheet A, while the C-terminus forms strand s1C in β-sheet C producing a 70Å separation of the P1 and P1’ residues inhibiting PAI-1/proteinase intereactions due to spatial distortion [15-17].

## PAI-1 in Vascular Pathology

PAI-1 is abundant in platelets; upon tissue injury, plasma PAI-1 levels increase approximately 10-fold likely as a consequence of platelet activation [18-20]. PAI-1 rapidly inhibits both tissue-type (tPA) and urokinase (uPA) plasminogen activators with second order rate constants approximating 3.5 × 107 M-1s-1 [14,21,22]. The primary role of the plasminogen activator system is to generate the active enzyme plasmin from its zymogen precursor, plasminogen, a key step in the fibrinolytic cascade [23-25]. Indeed, PAI-1 deficiency in humans results in a hyperfibrinolytic state and abnormal bleeding after trauma or surgery [26-30]. PAI-1 is a critical, rate-limiting, factor that impacts thrombosis, fibrin accumulation and ECM remodeling [31]. Inhibition of the fibrinolytic system by PAI-1 overexpression, moreover, has been implicated in various pathologies including tissue fibrosis, metabolic disorders and cardiovascular disease (i.e., atherosclerosis, vessel stenosis). A recent report, furthermore, highlights this causative relationship and provides evidence that a small molecule PAI-1 inhibitor (TM5441) confers protection to the development of cardiac hypertrophy, hypertension and periaortic fibrosis in L-NAME-treated mice [32,33].

**Figure 2:**
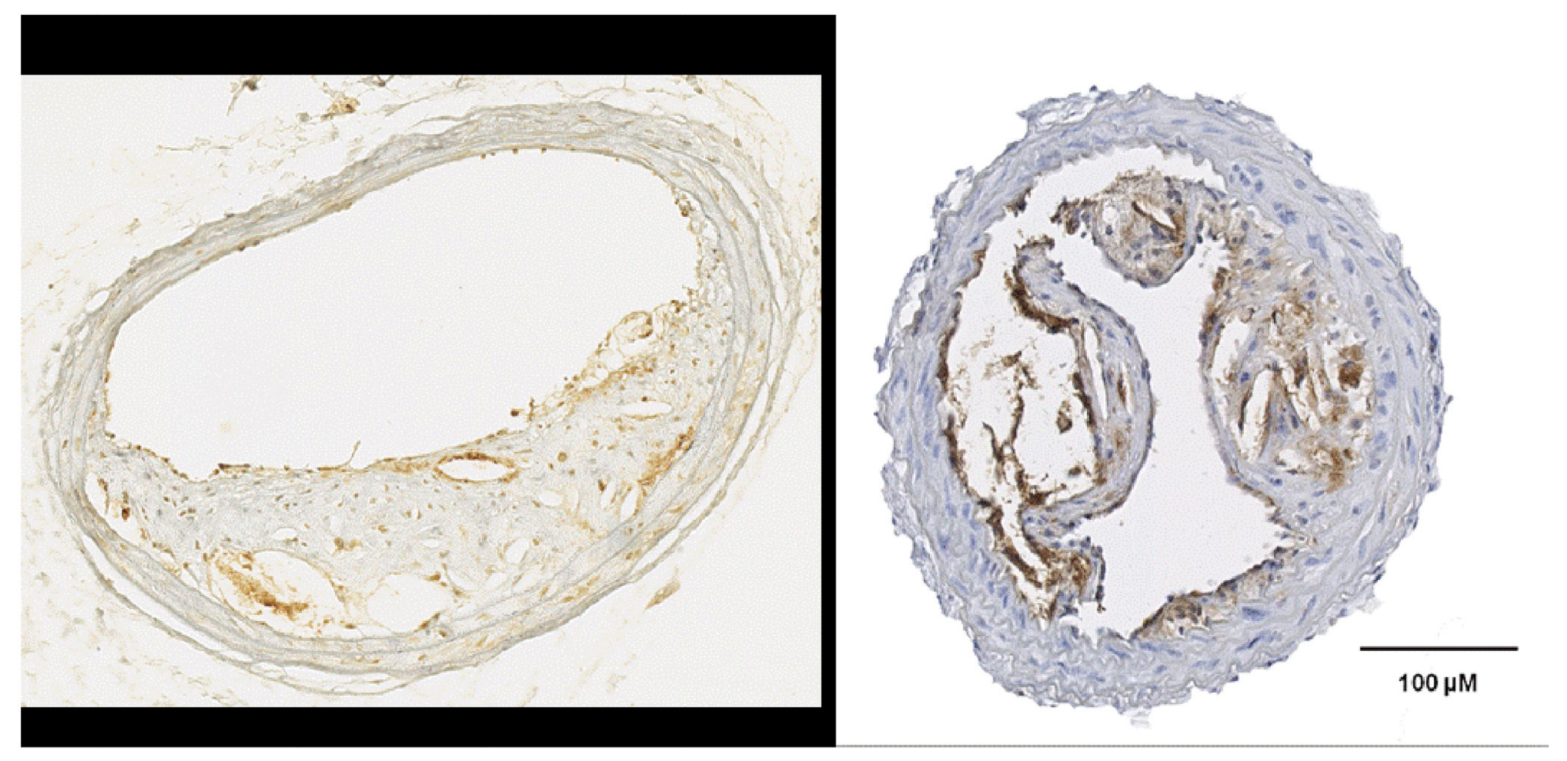
Induction of Carotid Plaques in Mice and PAI-1 Immunohistochemistry. In humans, atherosclerosis develops in areas of low shear stress and oscillatory blood flow. Placement of shear stress-modifying cuff or conical cast around the common carotid artery in mice mimics the disturbed blood flow characteristic of human carotid arteries [47,48]. Constriction promotes the formation of vulnerable plaques proximal to the device. In atherosclerosis prone ApoE−/− mice, carotid cuffing induces plaques with foam cells, necrosis, and thin fibrous caps that are hallmarks of vulnerable human plaques; ~30% of ApoE−/− plaques have intraplaque red blood cells, indicative of rupture [48]. Section on left exhibits a neointima with abundant PAI-1+ cells (brown deposits); right section illustrates a PAI-1 immunoreactive inflammatory plaque. One advantage of this model is the accessibility of the carotid arteries to ultrasound biomicroscopy, enabling longitudinal studies of plaque formation and providing an evaluation tool for the effects of drug treatment. Ultrasound may eventually be tuned to discriminate between stable and unstable human plaques. The details of the mouse carotid constriction model and blood flow characterization have been published [49]. All animal procedures were approved by the Albany Medical Center Institutional Animal Care and Use Committee (protocol # 912461) and carried out in compliance with NIH regulations. While both wild-type and genetically-engineered mice have proven invaluable in the implication of PAI-1 in cardiovascular and fibrotic disease, caution is warranted in extrapolating data from the various murine models of vascular injury to human disease since PAI-1 levels, both in plasma and platelets, are significantly lower in mice (discussed in [50]). These data illustrate the relationship between carotid cuff injury and PAI-1 expression as well as highlight the lack of a neointimal response to placement of copper cuffs around the carotid arteries of PAI-1−/− mice [51].

Atherosclerosis the first clinical association of increased PAI-1 with cardiovascular pathology was the finding of elevated plasma PAI-1 levels in young survivors of myocardial infarction (MI); PAI-1 levels were a significant risk factor for infarct recurrence [34,35]. PAI-1 increases precede MI and predispose patients to MI independent of other risk factors [36,37]. As infarct is caused by interrupted blood flow as a result of a ruptured vulnerable atherosclerotic plaque, correlations between PAI-1 and atherosclerosis resulted in the discovery of high PAI-1 levels in vascular lesions suggesting that this SERPIN plays an integral role in atherogenesis [38-40]. Importantly, atherosclerosis-prone apolipoprotein E-null (ApoE−/−) mice had a 3-fold up-regulation in plasma and smooth muscle cell PAI-1 mRNA in advanced atherosclerotic lesions compared to wild-type controls [41] suggesting a role in disease progression (e.g., Figure 2). As proof-of-concept, double deficient PAI-1−/−/ApoE−/− mice had decreased neointima formation after ferric chloride-induced arterial injury compared to PAI-1+/+/ApoE−/− controls [42]. There are, however, contrary data. PAI-1 deficiency augmented atherosclerotic progression in the ApoE−/− genetic background in one study [43,44] while a cross of LDL receptor-null, atherosclerosis-prone with PAI-1−/− mice did not change vessel lesion formation [44].

Complicating this issue, while atherosclerosis in the aorta of PAI-1−/−/ApoE−/− and PAI-1+/+/ApoE−/− were similar, there was a decrease in disease progression at the carotid bifurcation in PAI-1−/−/ApoE−/− suggesting PAI-1 may potentiate lesion development at sites of turbulent flow [45]. Since atherogenesis involves lipid accumulation, persistent inflammation, vascular injury, fibrin as well as ECM deposition, elevated PAI-1 expression and its major tissue injury-associated inducer TGF-⊠1 [3,46] it is likely the atherosclerotic response will vary as a function of vascular site, blood flow mechanics, type of injury, tissue levels of PAI-1 and TGF-β1, plaque vulnerability, genetic background and other disease co-factors.

**Figure 3:**
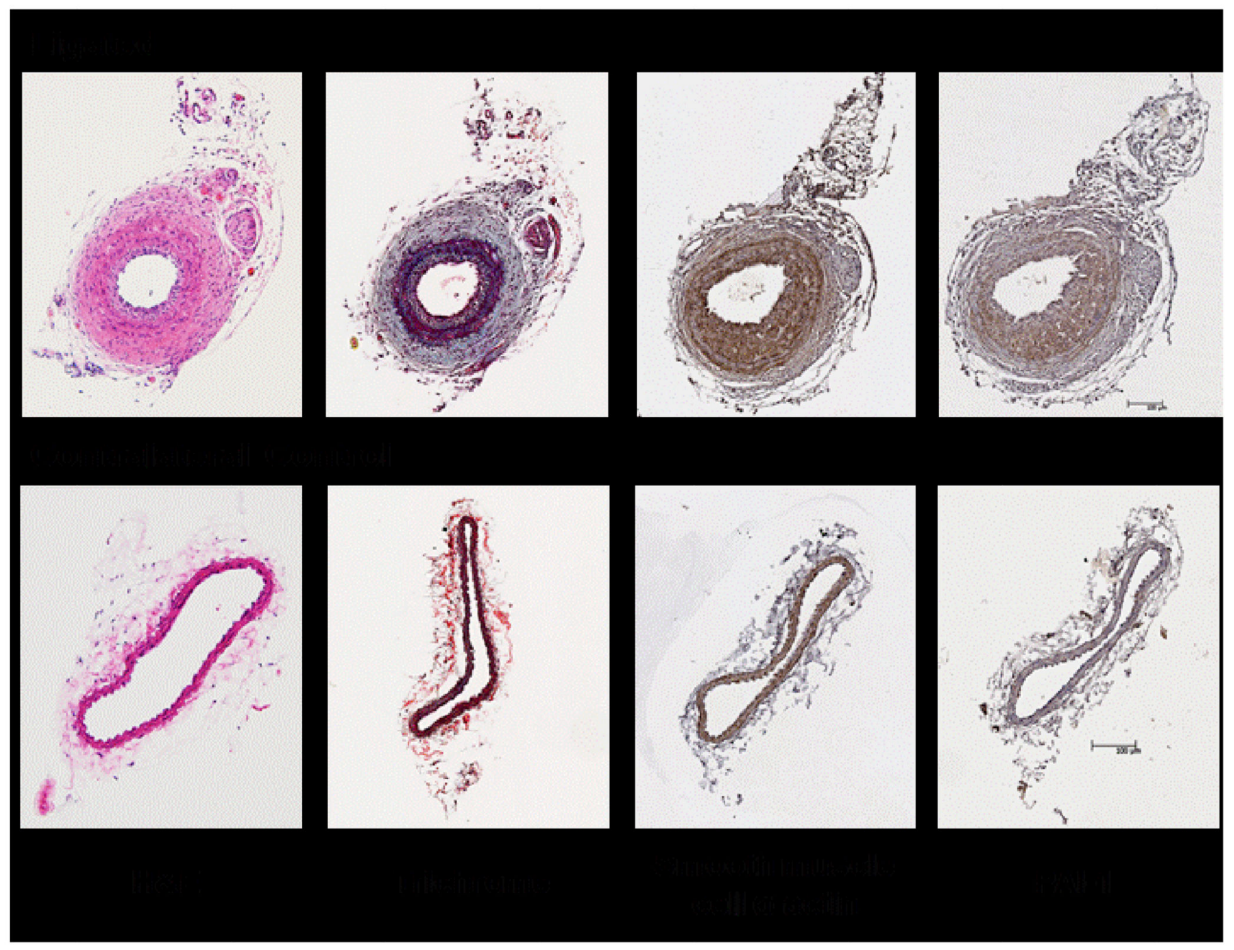
Carotid ligation-induced PAI-1 expression. FVB/NJ mice (Jackson Labs) were anesthetized by intraperitoneal injection of ketamine (0.1 mg/gm) and xylazine (0.01 mg/gm). Following incision site preparation, the left carotid artery was exposed with a small 8-10 mm midline incision in the neck and blunt dissection to free the left common carotid artery and branches from surrounding tissue. The common carotid artery was ligated proximal to the internal and external carotid bifurcation with a 6-0 sterile silk suture. The incision site was closed using 5-0 silk suture in a subcuticular pattern. Paraffin-embedded sections were stained with H&E as well as with Trichrome reagent; immunohistochemistry utilized antibodies to smooth muscle β-actin and PAI-1 as described [57].

Neointimal hyperplasia and stenosis. Currently, the main treatment for atherosclerosis is transluminal coronary angioplasty (with and without stent placement). This procedure often results in pathological remodeling and restenosis, a major limitation to clinical intervention. Treatment, and prevention, of in-stent restenosis have been disappointing. Drug-eluting stents, moreover, pose a significant danger of late thrombosis even after successful implantation [52]. Elevated PAI-1 mRNA/protein expression in the vascular wall adjacent to a thrombus was evident upon implantation of indwelling polyethylene tubing in rabbit carotid arteries [53]. Furthermore, adenoviral delivery of PAI-1 potentiated neointima formation after balloon-catheter angioplasty [54] while neointima formation was markedly attenuated following copper-induced arterial injury in PAI-1−/− mice [55]. Similarly, using a carotid artery ligation model, PAI-1 protein levels were elevated in neointimal lesions 14-days after occlusion (Figure 3, compare bottom left to bottom right). PAI-1 expression, furthermore, co-localized with smooth muscle α-actin suggesting synthesis by smooth muscle cells (Figure 3, top left). Importantly, in post-transluminal coronary angioplasty PAI-1 activity was significantly greater in patients with restenosis compared to those without recurrence [56].

## PAI-1 as a Regulator of Cell Migration: A Key Event in Vessel Stenosis?

PAI-1 modulates cellular migration in response to tissue or monolayer injury [58] likely as part of the grow-or-go dichotomy [59]. Medial smooth muscle cell migration and proliferation, followed by ECM synthesis, is a central mechanism in the development of vascular restenosis in post-angioplasty patients. TGF- β1, a prominent pro-fibrotic vascular factor, increases intimal expansion via smooth muscle cell migration in both injured and uninjured arteries and elevated levels of TGF- β1 are evident in human restenotic lesions [60-64]. TGF- β1 appears to stimulate neointimal development through upregulation of PAI-1 [3].

PAI-1 positively impacts cellular motility both through its anti-proteolytic and signaling functions. Receptor-engaged uPA and PAI-1 focalize to the leading edge of a migrating cell where they titer cell-ECM interactions, thereby regulating spatiotemporal deposition of matrix, providing a platform for cell migration [65,66]. Alternatively, PAI-1 can modulate cell locomotion by binding to vitronectin via RGD-dependent interactions which effectively stabilizes PAI-1 activity by extending its half-life while displacing vitronectin from its αvβ3 and αvβ5 integrin receptors [67].

Independent of its role in proteolysis, PAI-1 stimulates cell motility via interacting with low-density lipoprotein receptor-related protein-1 (LRP1) triggering Jak/Stat1 signaling [68]. The three conformations of PAI-1 (active, latent and cleaved) bind LRP1, activate the Jak/Stat pathway and drive migration [69,70]. PAI-1 also regulates the availability of cell-surface integrins by promoting their endocytosis in an LRP-1-dependent manner via PAI-1/uPA/uPAR (uPA receptor)/LRPI/integrin complexes. While PAI-1 and uPA are degraded, uPAR, LRP-1 and integrins are recycled back to the leading edge. This process fine tunes the special control of pericellular proteolysis and the overall cadence of cell detachment/re-adhesion required for efficient cell migration [71-73]. These data suggest that PAI-1 modulates cell motility under several contexts, both via anti-proteolysis and as a signaling molecule, particularly in the setting of increased injury-associated tissue TGF- β1 levels.

## Low Molecular Weight PAI-1 Antagonists

Several low molecular weight antagonists of PAI-1 are available [74] (Table 1). The first were diketopiperazines (XR330 and XR334) and the later, more potent, antagonists (XR1853, XR5082, XR5967, XR1121) which inhibit PAI-1 by inducing the transition from active PAI-1 to non-reactive PAI-1 [75-77]. Furthermore, by inhibiting the interaction of tPA/uPA and PAI-1 in a rat carotid artery thrombus model, XR334, XR5082 and XR1853 effectively increase fibrinolysis in vivo [76]. Similarly, AR-H029953XX and fendosalanthranalic acid derivatives of the known fibrinolytic antagonist flufenamic acid both inhibit PAI-1 by direct conversion into the latent, nonreactive conformation, but have not been tested in vivo [45-47].

The negatively charged antagonists ANS, bis-ANS and 1-dodecyl sulphurc acid and the positively charged XR-5118 overlap and localize around the flexible joint region of PAI-1, thereby inhibiting the RCL accessibility during interaction with proteinases [78-80]. This conformational rearrangement causes the reactive site to become inaccessible, thereby preventing tPA and uPA from binding and ultimately preventing the antiproteolytic capacity of PAI-1[80]. Of these, only XR-5118 has in vivo efficacy by increasing tPA activity and both reduced rat and rabbit arterial thrombus growth [81,82].

Recently, a class of polyphenolic compounds was found to inhibit PAI-1 with 10-1000-fold improved potency [83]. These compounds were found to reversibly interfere with the initial association of PAI-1 with its target protease, and two of the compounds (CDE-066 and CDE-081) showed efficacy in ex vivo plasma [83]. Only CDE-066 has in vivo efficacy, as evidenced by its ability to block PAI-1 activity in mice overexpressing PAI-1 [83]. Similarly, the PAI-1 inhibitor, IMD-1622 significantly reduced thrombi formation following rat arterial injury and inhibited neointimal formation in response to a murine copper-wire injury model [84]. Additionally, Tiplaxtinin and TM5007, indoleoxoacetic acid derivatives molecules which antagonize the activity of PAI-1 by inserting into the s4A position of β-sheet A as a mock molecule thereby inhibiting the PAI-1/tPA complex formation and have also been designed [85,86]. Importantly, both of these compounds, Tiplaxtinin and TM5007, are metabolically stable, non-toxic and showed good oral bioavailability and in vivo efficacy in a rat thrombosis model [85,87]. Tiplaxtinin, the most well studied low molecular weight inhibitor of PAI-1 has also been shown to attenuate asthmatic flare-ups, obesity, diabetes, cancer cell motility and angiogenesis in various studies, is the only low molecular weight antagonist of PAI-1 discussed here to be studied in a restenotic animal model [88-95]. It was observed that inhibition of PAI-1 with tiplaxitin caused significant reduction in Angiotensin II (Ang II)-induced medial, adventitial, and aortic wall thickening through a blood pressure dependent mechanism [95].

## Significance and Clinical Implications

Currently, the only treatment option is transluminal coronary angioplasty, a procedure that often results in substantial pathological remodeling, thus the predisposition of restenosis, and ultimately, considerable implications in cardiovascular health. Given the information regarding increased PAI-1 expression and activity in the development of neointimal hyperplasia, many efforts have been undertaken to develop pharmacological inhibitors of PAI-1 that may be clinical applicable down the road, however only Tiplaxtinin has been shown to reduce neointimal growth and found that Tiplaxtinin inhibits PAI-1 and TGF-β1-induced vascular smooth muscle migration and phosphorylation of Akt (not shown). As our data and others suggests, PAI-1 may modulate pathological remodeling of an injured vessel by promoting cell motility and survival. It is imperative that further research into these inhibitors of PAI-1 is underwent, in order to fully examine the role PAI-1 plays in vascular smooth muscle cell biology and pathogenesis and to determine the potential utilization of these small molecule inhibitors in pharmacological intervention and amelioration of neointimal lesions.

## Figures and Tables

**Table 1:** Mechanism of action

Mechanism of action	Designation	*In vivo*	Refs
Induces reactive center loop inaccessibility	1-dodecyl sulfuric acid	Not reported	[79,96]
	ANS	Not reported	[77,79,96,97]
	Bis-ANS	Not reported	[79,96,97]
	IMD-1622	Reduced rat arterial thrombus formation, and neointimal formation	[84]
	Tiplaxtinin	Reduced rat arterial thrombus growth, and reduced Ang II-induced medial, adventitial, and aortic wall thickening	[87,95]
	TM5007	Reduced rat arterial thrombus	[85]
	XR5118	Reduced rat arterial thrombus	[82]
Inactivating	AR-H029953XX	Not reported	[77,79,97]
	Fendosal	Not reported	[77]
	XR11211	Not reported	[75]
	XR1853	Reduced rat arterial thrombus	[76,77]
	XR330	Not reported	[78]
	XR334	Reduced rat arterial thrombus	[76,78]
	XR5082	Reduced rat arterial thrombus	[76,77]
	XR5967	Not reported	[98]
Inhibits initial interaction between PAI-1 and protease	CDE-066	Reduced PAI-1 activity	[83]
	CDE-082	Not reported	[83]
